# DISCOVER: a Data-driven Interactive System for Comprehensive Observation, Visualization, and ExploRation of human behavior

**DOI:** 10.3389/fdgth.2025.1638539

**Published:** 2025-09-19

**Authors:** Tobias Hallmen, Dominik Schiller, Antonia Vehlen, Steffen Eberhardt, Tobias Baur, Daksitha Withanage Don, Wolfgang Lutz, Elisabeth André

**Affiliations:** ^1^ Chair for Human-Centered Artificial Intelligence, University of Augsburg, Augsburg, Germany; ^2^ Department of Psychology, Division of Clinical Psychology and Psychotherapy, University of Trier, Trier, Germany

**Keywords:** human behavior analysis, computational models, data exploration, interactive system, behavioral indicators, psychotherapy, machine learning, multimodal analysis

## Abstract

Understanding human behavior is a fundamental goal of social sciences, yet conventional methodologies are often limited by labor-intensive data collection and complex analyses. Computational models offer a promising alternative for analyzing large datasets and identifying key behavioral indicators, but their adoption is hindered by technical complexity and substantial computational requirements. To address these barriers, we introduce *DISCOVER*, a modular and user-friendly software framework designed to streamline computational data exploration for human behavior analysis. *DISCOVER* democratizes access to state-of-the-art models, enabling researchers across disciplines to conduct detailed behavioral analyses without extensive technical expertise. In this paper, we are showcasing *DISCOVER* using four modular data exploration workflows that build on each other: Semantic Content Exploration, Visual Inspection, Aided Annotation, and Multimodal Scene Search. Finally, we report initial findings from a user study. The study examined *DISCOVER*’s potential to support prospective psychotherapists in structuring information for treatment planning, i.e. case conceptualizations.

## 1 Introduction

Understanding the nuances of human behavior is central to a variety of disciplines, from psychology and sociology to communication and computer science. However, the in-depth study of complex social interactions is often hampered by the limitations of conventional research methods. Traditional approaches often rely on manual data collection and observational methods, followed by detailed but time-consuming analysis. These processes require a significant investment of time, effort, and resources. These limitations often hinder the scope and depth of behavioral research and limit the ability to comprehensively explore complex phenomena.

Computational models that analyze key behavioral indicators such as social cues and interaction patterns have emerged as promising alternatives to traditional, labor-intensive methods. Despite their potential, numerous challenges still need to be addressed before these tools can be used on a large scale. Due to their high computational cost and technical complexity, they are difficult to access for researchers without technical expertise or specialized equipment.

To address these issues, we introduce *DISCOVER*,^1^ an adaptable open-source software framework specifically designed to facilitate computational methods for data exploration and analysis in behavioral research. The primary objective of *DISCOVER* is to democratize access to advanced computational tools, empowering researchers from a wide range of disciplines and levels of expertise to perform detailed and sophisticated behavioral analyses without requiring extensive technical knowledge.

This paper demonstrates the capabilities and versatility of *DISCOVER* through four illustrative workflows, each showcasing different aspects of data exploration and analysis:

1. **Semantic content exploration**: By integrating large language models directly into the framework, *DISCOVER* enables researchers to interactively explore and analyze the semantic content of spoken language during human interactions. This feature facilitates the extraction of meaningful patterns and insights from textual data with minimal effort.
2. **Visual data exploration**: *DISCOVER* uses integrated social signal processing tools to assess and visualize a variety of behavioral cues. These tools help to uncover and interpret patterns in behavioral data, providing a clearer understanding of social dynamics and interaction cues.
3. **Assisted annotation of behavioral cues**: *DISCOVER* aids in annotating meaningful behavioral indicators, streamlining what is typically a labor-intensive process. By automating and enhancing such annotation workflows, researchers can focus on higher-level analysis and interpretation.
4. **Multimodal scene search**: The framework also supports the automation of multimodal scene search within recorded sessions. This functionality allows users to locate and analyze key moments efficiently, significantly reducing the time required for manual review.

To demonstrate the workflows and analyses possible with *DISCOVER*, we conceptualized a user study for treatment planning in psychotherapy that can be conducted with (prospective) psychotherapists as participants.

## 2 Related work

Previous research on human-computer interaction has proposed to analyze multimodal features in specific conversational contexts to study human behavior. Over time, a variety of tools have been developed to annotate these behavioral cues in the context of their occurrence in human interaction. Prominent examples include ELAN (1), ANVIL (2), and EXMARaLDA (3). These tools offer layer-based tiers to link time-anchored labeled segments to an audio or a video signal, specifying start and end timestamps along with a label for each time period in the underlying signal. Think of bodily behaviors, like “waving a hand,” or mimic behavior such as “smiling” or “nose wrinkling.” We call these annotations *discrete* (4), if the set of labels is predefined, e.g., categorical emotions, and *free* for arbitrary labels, e.g., transcription from audio. *Continuous* annotations (4) on the other hand are labeled on a continuous numerical scale, allowing an annotator track and rate an observed stimulus over time, e.g., valence or arousal values rated on a continuous scale from 0 to 1. One of the first tools that allowed annotators to trace emotional content in real-time on two dimensions (activation and evaluation) was FEELtrace (5). Its descendant Gtrace (General TRACE) (6) allows the user to define their own dimensions and scales. More recent tools for performing continuous descriptions are CARMA (Continuous Affect Rating and Media Annotation) (7) and DARMA (Dual Axis Rating and Media Annotation) (8).

Recently, these tools have evolved to include automatic detection of behavioral cues and social signals, partially eliminating the need for manual data annotation. For example, Emodash (EMOtional DASHboard) (9) has been designed to improve tutors’ retrospective understanding of learners’ emotions. Based on automated facial expression recognition within a video conferencing learning setting, it also allows users to manually annotate scenes as positive or negative. REsCUE (REal-time feedback on behavioral CUEs) (10) aids coaching practitioners in detecting unconscious behavior of their clients. To this end, REsCUE uses an unsupervised anomaly detection algorithm to cluster multimodal data as posture and gaze and identify outliers. MultiSense (11) can assess a person’s affective state by inferring various indicators from audio-visual and depth input signals. The tool focuses on its application within the mental health domain to assess indicators of psychological distress such as depression or post-traumatic stress disorder. MeetingCoach (12) is an AI-driven feedback dashboard designed to enhance the effectiveness and inclusivity of video-conferencing meetings by providing both personalized and shared insights into meeting dynamics, such as engagement summaries of participants or speaking turn and speaking time distributions. MACH (My Automated Conversation coacH) (13) is a virtual agent that responds in the context of job interview training to human behavior in real-time by applying facial expression, speech, and prosody recognition. Afterwards, visual feedback is provided, helping to improve social skills. The ConAn (CONversation ANalysis) (14) has been developed with a focus on group conversation analysis. To this end, it automatically analyzes the gaze behavior, body movement, speaker activity, and facial expressions of participants’ using a single $360^{\circ }$ camera.

These visualization-based methods were developed with specific goals and target groups in mind. As a result, the choice of features to be displayed is usually tailored to this specific use case. Therefore, these solutions suffer from a lack of customizability that prevents users from adapting the features to their individual needs.

The SSI framework (Social Signal Interpretation) by Wagner et al. (15) presents an alternative approach, by implementing a modular, multimodal signal processing pipeline, facilitating both online and offline recognition tasks. The plug-in system within SSI allows users to develop custom modules and integrate them into the processing pipeline. Similar to SSI the opensense (16) platform has been designed to facilitate real-time acquisition and recognition of social signals through multiple modalities. It also follows a modular pipeline design and builds on Microsoft’s Platform for Situated Intelligence (17), which enables the processing of human behavioral signals and supports various sensor devices and machine learning tools. Barz et al. (18) developed the MultiSensorPipeline, a lightweight and adaptable framework for creating multimodal-multisensor interfaces using real-time sensor data. While the framework is conceptually similar to SSI and OpenSense it focuses on a concise set of concepts and functionalities that facilitate the creation and execution of complex processing pipelines. Implementing specific modules is left to the user, making the tool better suited for developing prototypes of custom multimodal-multisensor processing pipelines than for using standard modules to analyze social signals. In contrast to the pure visual exploration of data, Providence (19) implements a scene search approach to investigate social behavior in multimodal data. Hereby, a human analyst can formulate queries containing non-verbal (e.g., nodding, facial expressions), linguistic (e.g., sentiment), or para-linguistic (e.g., speech speed or volume) to search for specific scenes in a human conversation. Providence automatically extracts the respective features required by a query and searches for scenes fulfilling the specified conditions in the data.

Although the approaches presented have their respective advantages and disadvantages, there are two common shortcomings: Efficient use of computing resources and a compromise between flexibility and complexity. Except for Providence, all the tools introduced are solely intended for operation on local machines. This leads to inefficient use in multi-user scenarios, in which users either have to take turns on a local machine or each user needs their individual workstation that is capable of running such tools. Considering hardware demands and energy consumption of state-of-the-art machine learning models, this issue extends beyond financial concerns to ecological ones. Further, it becomes evident that off-the-shelf solutions have constraints in their applicability, whereas more adaptable approaches necessitate greater technical proficiency, thereby posing a barrier to entry.

## 3 Framework

In this section, we introduce *DISCOVER*, a software framework designed to facilitate the study and analysis of human behavior, with a focus on social signal processing. Current tools are often challenging to modify for new data formats or study topics, and they frequently call for a high level of programming expertise. To overcome these constraints, *DISCOVER* offers a platform that is extensible and modular for researchers with different levels of technical proficiency.

**Figure 1:**
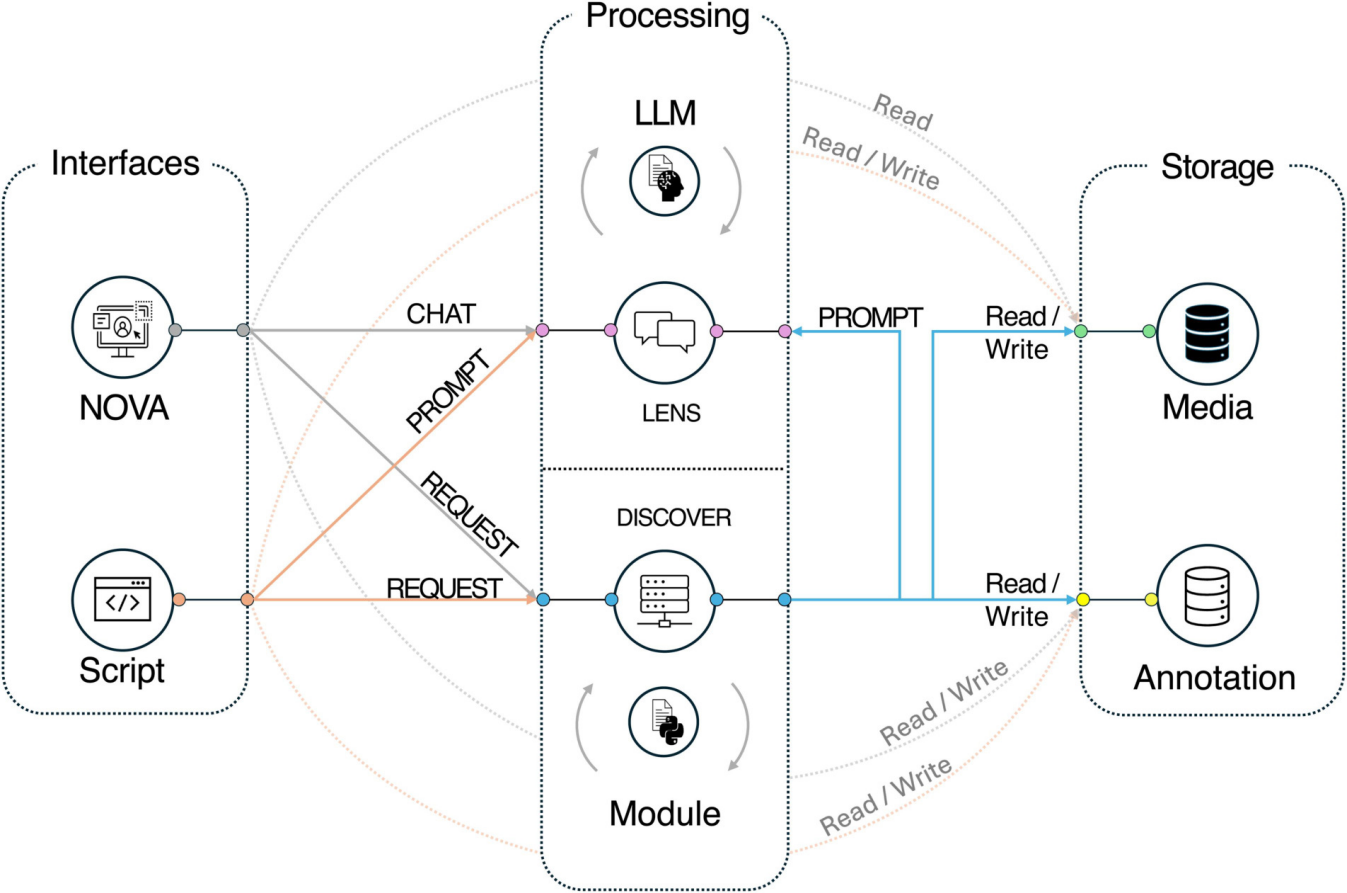
Overview of the system components for the *DISCOVER* framework.

The modular design of *DISCOVER*’s architecture prioritizes ease of maintenance, scalability, and flexibility. An overview of the system architecture is presented in Figure 1. The key elements and their features are explained in detail in the following subsections.

### 3.1 User interface

The *DISCOVER* framework focuses on accessibility for researchers with different levels of technical knowledge. While a REST API allows experienced users to interact directly with the framework and use its functionalities via scripts in any programming language, the primary user interface is provided by the open-source annotation tool *NOVA*^2^ (20). The integration of *NOVA* as a user interface enables researchers without programming skills to effectively explore and analyze data within *DISCOVER*.

*NOVA* extends traditional annotation processes by integrating cooperative machine learning (CML) and explainable artificial intelligence (XAI) capabilities. This integration provides annotators with seamless access to automated model training, prediction capabilities and advanced explanation algorithms directly in the user interface, enabling iterative refinement of annotations and deeper insights into the data.

**Figure 2:**
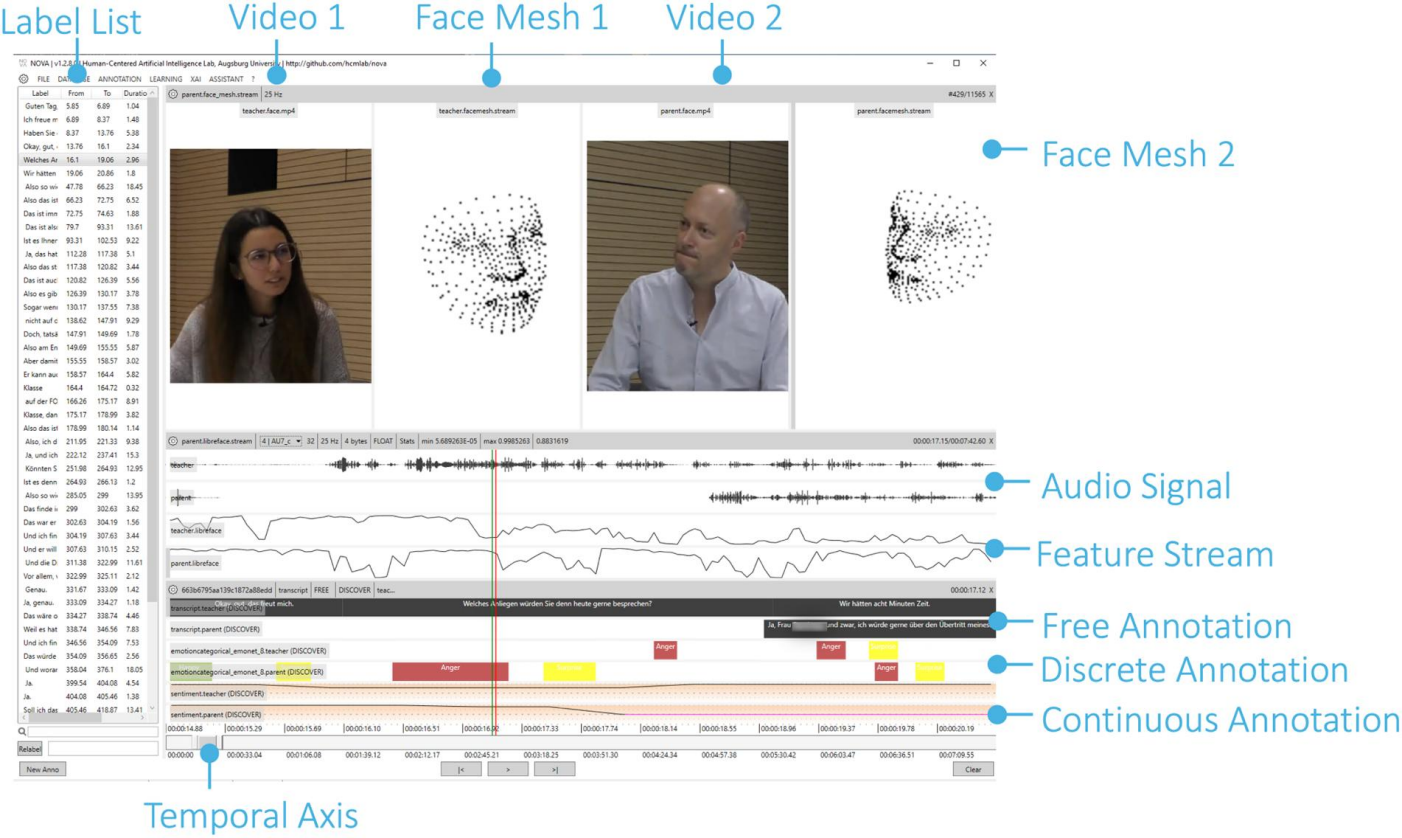
*NOVA* allows to visualize various media and signal types and supports different annotation schemes. From top to bottom: upper-body videos along with face tracking, audio streams of two persons during an interaction, and activation of action units (feature stream). In the lower part, free-value (transcription), discrete (emotions), and continuous (sentiment) annotation tiers are displayed. In a *DISCOVER*-heavy use, only the raw videos and audios are manually provided, everything else is automatically extracted. In a mixed use, you can manually annotate own defined defined schemes, e.g. discrete annotation for smiling.

The *NOVA* UI is specifically designed for the annotation of long, continuous recordings involving multiple modalities and subjects (see Figure 2). The interface supports an unlimited number of synchronized media tracks—including video, audio, facial features, skeleton data, and depth images—allowing for a comprehensive multimodal analysis. Below the media tracks, users can create and manage multiple annotation tracks representing different annotation types (discrete, continuous, and free-text transcription).

Beyond basic annotation, *NOVA* provides functionalities for processing annotations from multiple annotators (human or machine). This includes calculating inter-rater agreement using statistical measures such as Cronbach’s α and Cohen’s κ, which help assess the reliability and consistency of the annotations. These features contribute to the framework’s goal of facilitating collaborative and rigorous data analysis.

### 3.2 Annotation data storage

To facilitate a collaborative and scalable annotation process, *DISCOVER* utilizes the *NOVA* MongoDB database^3^ as its back-end storage. MongoDB’s flexible schema allows for the storage of diverse annotation types and facilitates the integration of annotations from various sources.

The database allows multiple users to simultaneously load, save, and track annotation progress, fostering real-time collaboration.

Beyond supporting human annotators, the database also serves as an interface for “machine users”—automated agents or algorithms that can create and access annotations. This allows for the integration of automated annotation tools and facilitates iterative refinement of annotations through human-machine collaboration. For example, machine users can pre-label data or suggest annotations for human review.

The database design supports the dynamic addition of new annotators, annotation schemes, and data sessions without requiring significant modifications to the system, ensuring long-term adaptability and scalability.

Beyond the integration of the *NOVA* MongoDB, *DISCOVER* offers flexible output options. Annotated data can be directly written to a specified data directory for persistent storage and subsequent analysis. Alternatively, the framework can return annotation files as a response to an API call, enabling real-time integration with other applications and workflows. This flexibility allows users to tailor the output method to their specific needs and application requirements.

### 3.3 Media file storage

To efficiently manage large media files associated with annotation tasks, *DISCOVER* leverages a data storage component that follows the structure of the open-source cloud hosting framework Nextcloud.^4^ Media files, including raw recordings and derived features (e.g., facial landmarks, skeleton points, speech characteristics), are stored separately from the annotation database. This separation is crucial for performance, as continuous data streams - such as hours of recordings of two interlocutors in 4 K at 60 Hz—can quickly generate significant storage requirements. Offloading these large files to dedicated file sharing servers relieves the database of significant I/O overhead and improves the overall responsiveness of the system.

### 3.4 Processing server

The processing server functions as the central computational engine for data processing within the *DISCOVER* framework. It implements a lightweight web server, exposing a REST API that facilitates interaction with the annotation and data storage components to extract meaningful features from the data such as sentiment scores, facial expression analysis, or coded behavioral events. These features are then readily available for visualization within the *NOVA* user interface or for subsequent, more complex processing.

Exposing a REST API provides several key advantages. It enables not only access from the *NOVA* UI but also programmatic access via scripting languages like Python and R. This low entry barrier allows researchers to rapidly prototype and implement custom analyses, while seamlessly integrating the processing functionality into automated workflows. The API supports access to annotated data, raw media files, and intermediate processing results.

By combining computational demands onto a single, potent machine, the server-based architecture also encourages effective resource utilization. This method lowers both the initial investment and continuing maintenance costs by minimizing the need for individual researchers to purchase and maintain specialized hardware. Through resource pooling and the utilization of the server’s processing power, *DISCOVER* optimizes hardware efficiency and enables scalable analysis without necessitating corresponding infrastructure expansions. For computationally demanding tasks like video processing and machine learning, where multiple users can efficiently use dedicated GPUs or specialized processors, this shared infrastructure model is especially advantageous.

Furthermore, the standardized input and output formats of the processing modules facilitate the sharing and reuse of extracted features and predicted labels.

### 3.5 LENS

Recent advances in large language models (LLMs), such as ChatGPT^5^ and Llama (21), have demonstrated significant potential for enhancing textual analysis capabilities, such as text summarization (22–24), sentiment analysis (25, 26), and argument mining (27, 28). To leverage these capabilities within the *DISCOVER* framework, we have developed LENS (Learning and Exploring through Natural language Systems), a component designed to integrate a variety of LLMs into the infrastructure.

Fundamentally, LENS is a lightweight web server providing a unified API for accessing LLM functionality. This API abstracts the underlying model implementation, allowing requests to be routed to either external service providers (e.g., OpenAI) or self-hosted models (e.g., via Ollama^6^). Through seamless integration with the *NOVA* user interface, users can interact with AI-powered assistants via a chat interface to analyze textual data, such as dialogue transcripts.

The modular design of LENS allows users to dynamically switch between available LLM services based on performance characteristics and privacy considerations. Furthermore, the output from LENS can be directly integrated into the annotation process, enabling automated or semi-automated annotation of textual data—for example, utilizing an LLM to annotate transcripts with sentiment labels (negative, neutral, positive).

## 4 Modules

*DISCOVER* relies on exchangeable modules to infer behavioral indicators from recorded data. Each of these modules can be understood as a configurable building block, consisting of predefined inputs and outputs with module-specific options. *DISCOVER* is fully extensible with custom modules, that can be shared with others through open-sourcing.^7^ However, to keep the entrance barrier low and provide value for a coding-averse target group we also provide a number of ready-to-use processing modules. The following section provides an overview of the currently integrated modules.

### 4.1 Audio

When analyzing human speech to gain behavioral insights, one needs to distinguish between the verbal and vocal components of speech. Verbal refers to communication that is expressed in words or language. It involves the use of language to convey ideas, thoughts, or information. Paraverbal on the other hand refers to the sounds produced by the voice or the act of speaking. In the context of communication, “paraverbal” can refer to the tone, pitch, volume, and other qualities of speech.

#### 4.1.1 Verbal

A prerequisite to the analysis of the verbal content of spoken language is the conversion from speech to text (STT). STT-Systems have been an active area of research for decades. For the implementation of our STT module we rely on WhisperX (29), an adaptation of Whisper (30), that provides improved timestamp accuracy, support for longer audio sequences, and faster transcription performance.

#### 4.1.2 Speaker diarization

The available datasets are typically recorded with the focus of manual human analysis rather than automatic processing. A common example of this is the recording of a single audio signal for several speakers. While it is a mostly trivial task for a human listener to distinguish between the voices of speakers and map the content of the spoken language to the respective person, this information gets lost during the STT process. To account for this loss of information *DISCOVER* implements a speaker diarisation module, which maps segments of a multi speaker dialogue transcript to individual speakers. To this end we rely on Pyannote (31, 32) and SpeechBrain (33) to embed and cluster voiced segments in the audio signal unsupervised and agglomerative. We can also leverage data exploration using an oracle approach to assign those clusters to individual speakers, by providing reference speaking terms within the audio signal.

#### 4.1.3 Sentiment analysis

Sentiment analysis is the process of computationally determining the emotional tone behind a piece of text, i.e., whether it is positive, negative, or neutral. It can be a valuable tool for analyzing human behavior, as it can provide a deeper understanding of individuals’ emotions and opinions. To enable the automatic prediction of sentiment, *DISCOVER* currently integrates two approaches. A multi-lingual (34) one, which accepts any BERT-like model string from HuggingFace,^8^ and a German language specific (35) model.

#### 4.1.4 Paraverbal

Speech emotion recognition (SER) refers to the task of automatically detecting and interpreting emotions conveyed through speech. It involves analyzing various acoustic features, such as pitch, intensity, and rhythm, to infer the underlying emotional state of the speaker. *DISCOVER* integrates a pre-trained model, proposed by Wagner et al. (36) to predict valence, arousal, and dominance values from a human voice.

### 4.2 Video

When investigating human behavior, hypotheses often include posture, gesture, and mimics. To this end, the video recordings mostly show upper body or wide shots. These sometimes need preprocessing like tracking and cropping. To do preprocessing and to predict aforementioned features, we provide a set of already implemented modules.

#### 4.2.1 Pose detector

Detecting body key points allows for downstream analyses like movement energy computation or gesture tracking. To this end, we implemented a module relying on the model BlazePose by Bazarevsky et al. (37). It is a lightweight convolutional neural network that is designed for mobile device usage.

#### 4.2.2 Face bounding box detector

The automatic derivation of behavioral indicators from a human face usually requires the localization of the facial area in an image or video. To this end, we rely on the BlazeFace model proposed by Bazarevsky et al. (38). The BlazeFace model is a lightweight face detection model that has been developed to run on mobile devices and thus requires only a minimum of computational resources to achieve super-realtime performance.

#### 4.2.3 Facial landmarks and meshes

For the further processing of the localized image, it is a common procedure to align facial images based on localized landmarks (39). Facial image alignment involves geometric transformations like translation, rotation, and scaling to convert the input face image into a standardized form. To this end, we employ the face mesh model by Kartynnik et al. (40), which infers an approximate 3D mesh representation of a human face from a single camera.

#### 4.2.4 Facial action units

Facial action units originate from an anatomical examination of the face and can be categorized according to the Facial Action Coding System (FACS) outlined by Ekman and Friesen (41). For automatic action unit detection and intensity estimation, we integrated the LibreFace (39) framework which achieves state-of-the-art performance in both tasks while improving inference times over other methods.

#### 4.2.5 Facial expression

Facial expression analysis involves automatically detecting subtle movements in facial muscles and identifying typical facial displays. The recognition of these expressions yields valuable insights into users’ social and emotional states. To facilitate robust facial expression prediction we integrated multiple models into *DISCOVER*: EmoNet (42), Relevance-Based Data Masking [RBDM; (43)], and LibreFace (39).

### 4.3 Multimodal feature extraction

Besides the above-mentioned modules, which directly provide insights about important indicators for human behavior to a user, *DISCOVER* also implements modality-specific feature extraction modules, that can be used to train custom detection models.

#### 4.3.1 Video

For the video modality we use the DinoV2 pretrained vision transformer models (44, 45) to extract features. Those models are pretrained in a self-supervised manner on a large dataset of 142 million images. As a result, DINOv2 models have demonstrated robust performance beyond training data, delivering usable general-purpose features without the need for fine-tuning.

#### 4.3.2 Audio

For the audio modality we rely on a pretrained w2v-BERT 2.0 encoder (46). Similar to the DinoV2 model, this model was trained unsupervised on a large data set of 4.5 million hours of audio, and demonstrates excellent performance for a variety of downstream tasks like speech to text, or expressive speech to speech translation. However, it is recommended to fine-tune the w2v-BERT 2.0 model before using it for a downstream task. Since this might not be feasible for technical unsavvy users we also integrated the openSMILE library (47), which extracts various handcrafted feature sets for the audio domain. Specifically, the GeMaps feature set (48) has been developed for general voice research and affective computing tasks and provides a good starting point for any speech-related classification task.

#### 4.3.3 Text

When it comes to extracting features from text representations, the language of the text is a necessary consideration. Since it is a key aspect of our framework to be employable in versatile scenarios across multiple languages, *DISCOVER* integrates a multilingual textual feature extraction using the XLMroBERTa (XLM-R) model by Conneau et al. (49). This model consists of a transformer-based architecture, trained on vast amounts of multilingual data crawled from the internet. In their experiments, the authors analyzed the capabilities of XLM-R for several tasks, including name entity recognition, cross-lingual question answering, paraphrasing, and sentiment analysis. The reported results indicate that the model performs close to or even better than comparable monolingual models for languages where vast training resources are available. Furthermore, the model showed substantial improvements over other state-of-the-art models across all tasks on languages, which are heavily underrepresented in datasets.

## 5 Interactive data exploration

**Figure 3:**
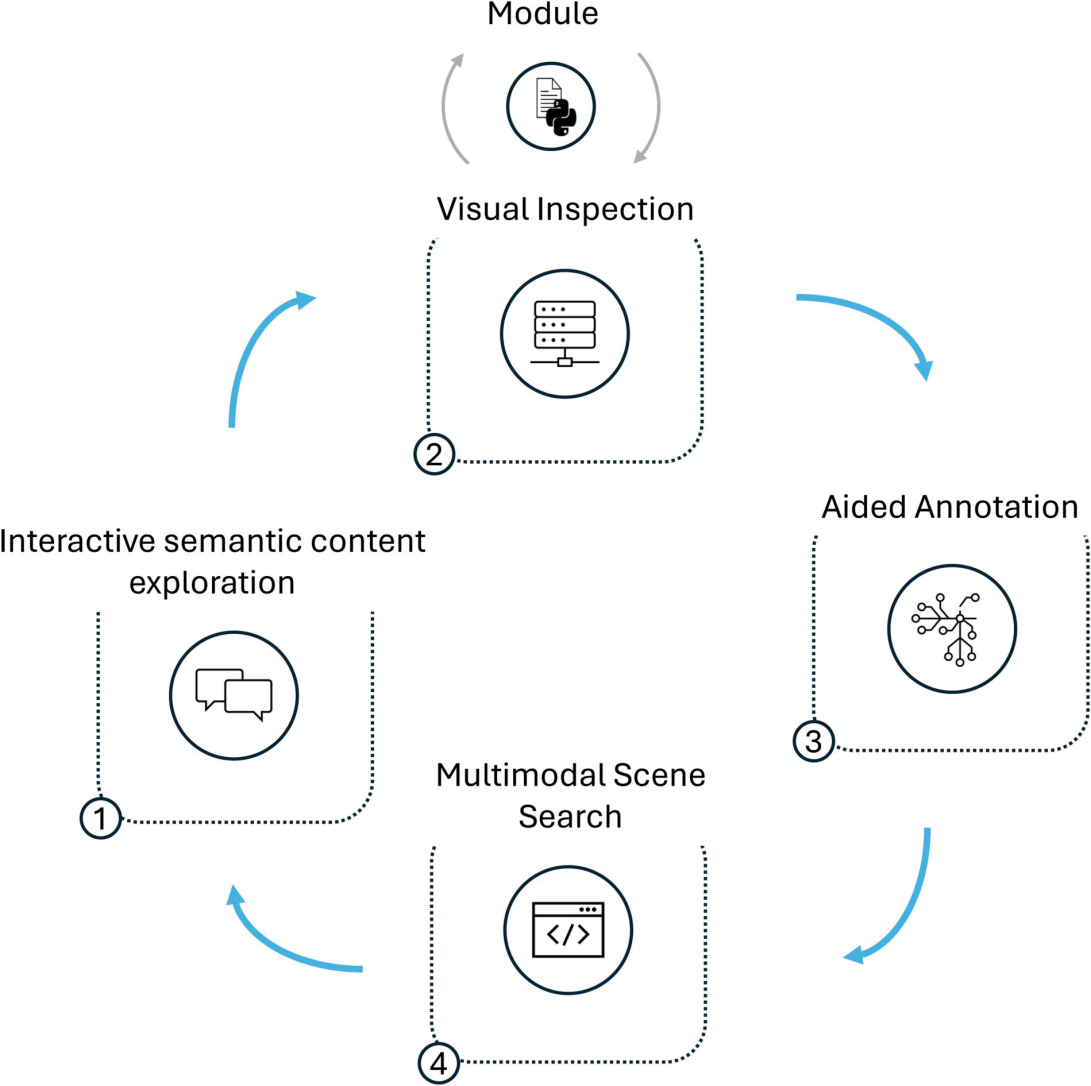
Schematic representation of the exploratory data analysis workflow. **(1)** Explore the content of a conversation using *LENS*. **(2)** Compute and visualize behavioral cues. **(3)** Annotate additional indicators with the help of cooperative machine learning. **(4)** Identify scenes that consist of an interplay of several indicators. Every step in the workflow can be repeated and adapted as necessary.

To illustrate the capabilities of *DISCOVER*, we present four exemplary workflows designed to examine unseen data and facilitate the discovery of new insights. While each of our showcases is building upon the results of the previous one, every workflow can also be carried out independently of the others. The complete iterative data exploration pipeline is depicted in Figure 3.

We demonstrate how our workflow operates by utilizing recordings of interactions between teachers and parents as applied in the work of Hallmen et al. (50). These videos are captured to evaluate the communication abilities of aspiring teachers in consultative situations and offer them constructive feedback. Within this context, the teacher is a trainee, while the role of the parent is portrayed by an actor. The subject of the discussion revolves around the child of the parent, who is facing challenges in school. For a translated version of the German discussion see Supplementary Material Section 1. The following use case is an exemplary workflow for a user in the role of a data analyst.

### 5.1 Semantic content exploration

As a first step towards finding indicators of communicative quality in the recorded interactions, a user wants to get familiar with the data and the task. To facilitate those tasks, *LENS* enables the interactive exploration of the semantic content of the dialogue. To start *LENS*, the user must first apply the WhisperX processing module to create a temporally aligned transcript of each speaker from the recorded audio signals.

**Figure 4:**
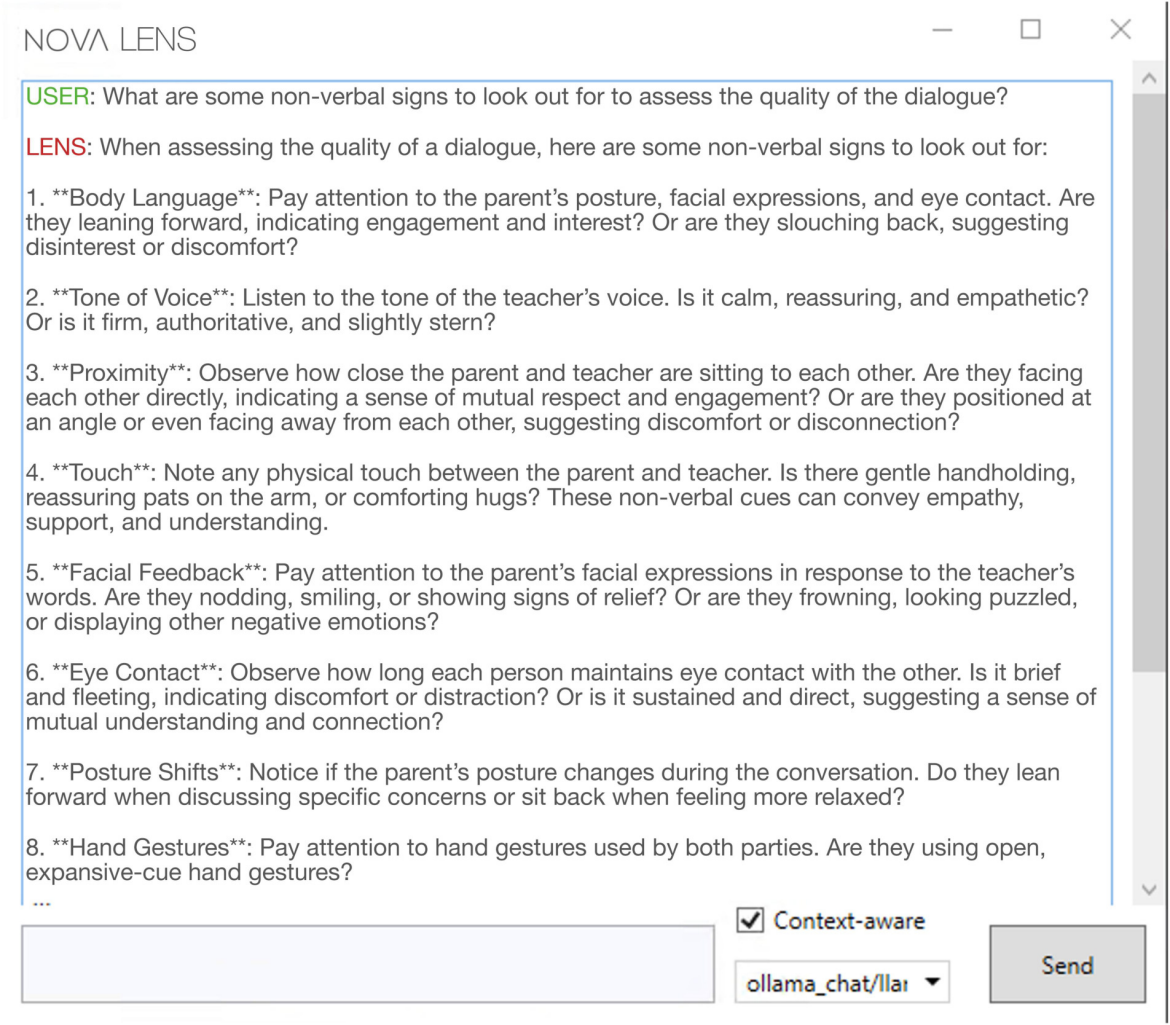
A user engages with *LENS* to explore the semantics of a conversation interactively.

After loading the transcription data into *NOVA* the user clicks on the *LENS* tab and a chat window opens that establishes a connection with *LENS* (see Figure 4). By selecting the “Context-Aware” checkbox located at the base of the screen, the transcript that has been loaded into *NOVA* in advance will be automatically transmitted to *LENS* as context for each message. That way a user can directly ask *LENS* any questions regarding the semantic content of the interaction. First, the user requests a summary of the interaction. *LENS* replies with a concise summary of the transcript, providing the user with information about the general setting and topic of the dialogue, the roles of the interlocutors, and the course of the conversation. As the user has gained those insights about the data, the next step is to gather more information on relevant behavioral aspects to look out for. To this end, the user asks directly about important criteria to assess the quality of communication in parent-teacher conferences. *LENS* answers by providing ten indicators for the assessment of dialogue quality, like *Positive outcome* or *Collaborative approach*. Each point is accompanied by a short description to clarify the meaning. From the previous summary, it is already clear that the teacher and parent are working together and the outcome of the dialog is positive as both parties agree on how they want to proceed in the future to support the child’s learning. After the initial summary, the user asked for a more detailed evaluation of the transcript, which focused on the previously identified indicators in order to gain more differentiated insights. In response, *LENS* provides further information regarding indicators such as *Empathy* and *Active Listening*, based on the full transcript. Finally the user also asks about nonverbal indicators of communication quality, *LENS* presented a list of significant findings, including *Facial Expressions*/*Smiles*, *Gaze* or *Vocal Inflections and Tone*. See Supplementary Material Section 2 for the complete dialogue between the user and *LENS*.

### 5.2 Aided annotation

The processing modules described in Section 4 are a core aspect of *DISCOVER*. However, they largely depend on the availability of pre-trained models for processing. Depending on the use case there might be no fitting model available for the task that the user is looking for. To alleviate this problem, *DISCOVER* provides support for feature extraction, that can be used to train custom models directly from within the *NOVA* user interface. To this end, *NOVA* implements a cooperative machine learning workflow that consists of the following 5 steps: Initially, the model undergoes training (Step 1) and is then utilized to predict unseen data (Step 2). Following this, an active learning module determines which portions of the prediction necessitate manual review by human annotators (Step 3). Subsequently, those labels are then reviewed by a human and corrected if necessary. Finally, the initial model is retrained using the updated labeled data (Step 5). This iterative process continues until all data is annotated. By actively integrating human expertise into the learning process, we enable interactive guidance and enhancement of automatic predictions. Following the suggestions of *LENS*, the user trains a new model that is able to detect smiles, based on the extracted face mesh data. Before continuing the next step the model is used to detect all instances of smiles for the teacher as well as the parent.

### 5.3 Visual inspection

During this stage, users visually examine sessions and their timelines to identify patterns and formulate hypotheses regarding underlying phenomena. To confirm or question these hypotheses, users can select a processing module, see Section 4, directly from the *NOVA* User interface and start an extraction job on the processing server. Once the processing is done, the results can be directly visualized in the user interface. This iterative process can be repeated as often as necessary for different modules. Since the results of the previous modules are stored either in the annotation database or on the media storage, the user can reuse them at any time, without recomputing them. To provide a clearer illustration of this process, we pick up on the previous example. Building upon the results of the semantic content exploration described in Section 5.1 the next steps are analyzing the non-verbal cues from the audio and the video signal and finding additional semantic indicators to assess the quality of communication based on the transcript.

**Figure 5:**
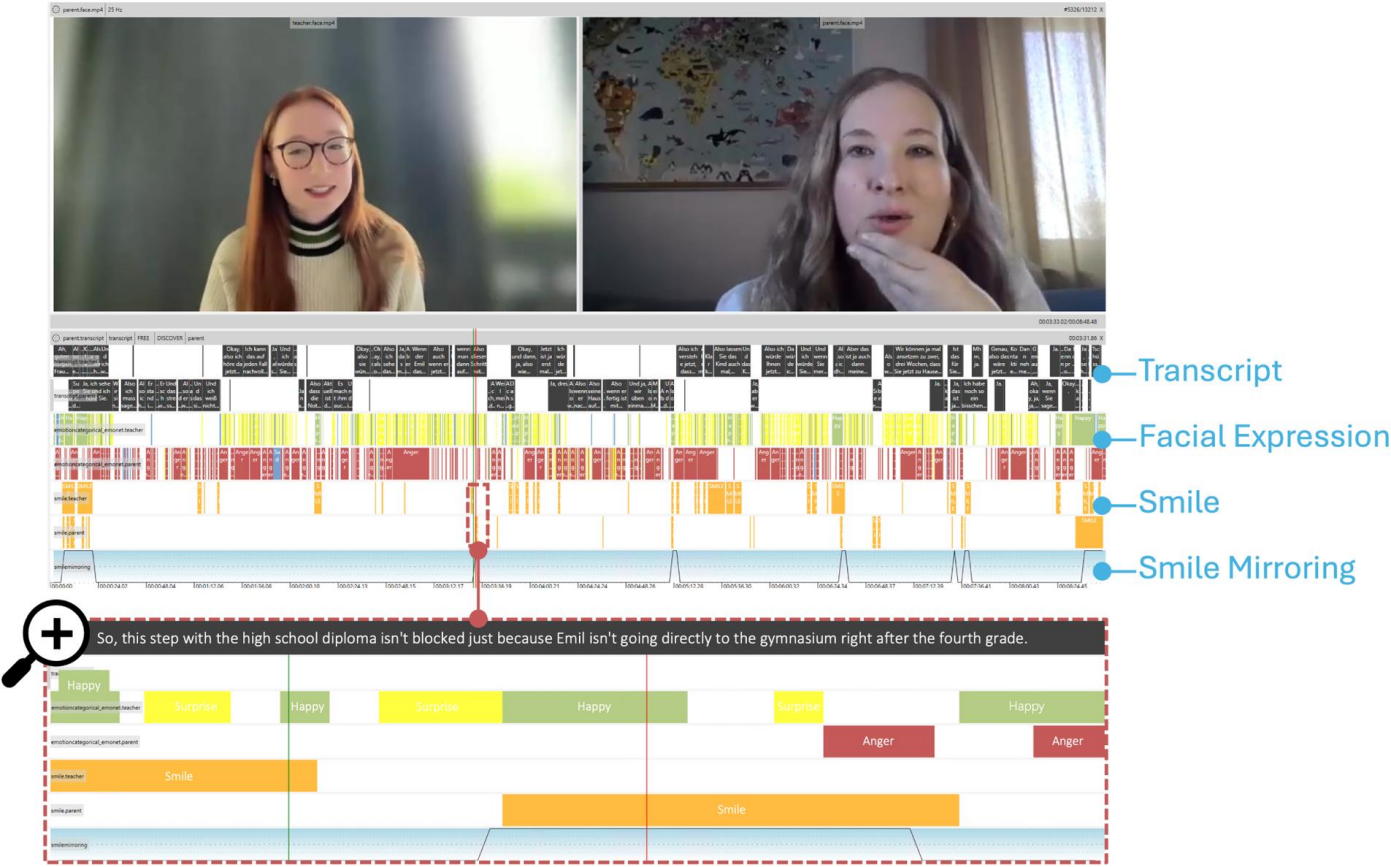
Visual data inspection with *NOVA* and *DISCOVER*. The session overview provides a comprehensive summary of the extracted behavior indicators across the entire session using pretrained models in *DISCOVER* for extracting transcript, facial expressions, and smiling. The user is exploring the data for ares of interest using *NOVA*’s synchronized play back of all streams and annotations. To preselect these areas, the user writes a script for temporally smoothed smile mirroring detection (Figure 6). Afterwards the user zooms in to such a smile-mirroring-scenne at the bottom, enabling a detailed analysis of identified scenes of interest.

To continue the exploration, the user utilizes the EmoNet module (see Section 4.2.5) to predict facial expressions exhibited by both the teacher and the parent. Upon loading the model’s predictions into *NOVA*. As depicted in Figure 5 it becomes apparent that the teacher’s facial expressions predominantly oscillate between “happy” and “surprised,” whereas the parent’s expressions tend to skew towards “anger” or “sadness,” although it is noted that anger may not always be accurate as the parent might simply be displaying a serious expression. In addition, the user now loads the smile annotation generated in the previous step, see Section 5.2.

Smiling occurs notably more often at the beginning and end of the conversation for both roles, with the teacher exhibiting smiles more frequently throughout the conversation. As the increased number of smiles at the beginning and end can likely be attributed to formal politeness the user first focuses on the middle sections of the conversation. Especially visually identifying, mirroring behavior where the smile of one interlocutor is mirrored by the other one, provides interesting insights. For example, there is a notable scene in which the parents’ smiles mirror the teacher’s smile, a moment in which the parents receive new and helpful information, indicating active participation in the dialog.

This observation underscores the significance of integrating visual and verbal cues to capture the dynamics of communication and emotional expression within the interaction.

### 5.4 Scene search

**Figure 6:**
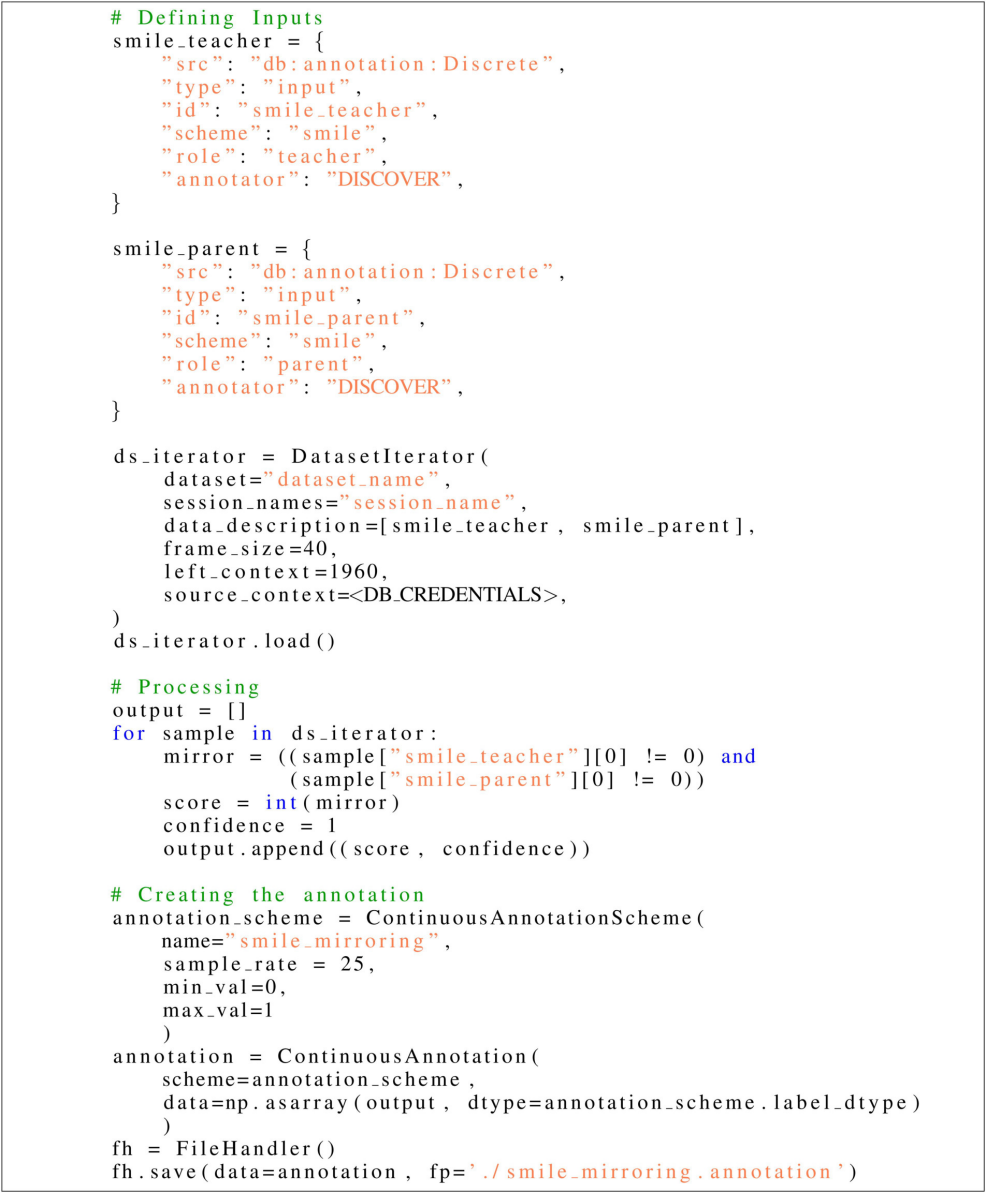
Sample code snippet demonstrating how to create an annotation for detecting smile mirroring. In this stage it is an iterative process of refining small scripts until one’s need is satisfied. Then the script can be expanded by *DISCOVER*’s interface with paramaters exposed for easy configuration and job submission from within *NOVA*, turning it into a module applicable on all different kinds of other sessions.

As shown, the exploration of conversational scenes through the analysis of interaction partners’ multimodal behavior presents considerable promise for investigating social dynamics. The process of visual inspection provides an effective method of identifying the constellation of social cues inherent in a specific scene. However, it is of limited use when it comes to retrieving all instances of such scenes in a recording session. First of all, it is easy to overlook certain scenes when scrolling through the timeline of longer recording sessions. Second, the analysis of social cue constellations that require precise assessment of either timings, e.g., the immediate facial expression to an event, or values, e.g., the degree to which an action unit is activated, can not be efficiently performed this way. Lastly, it is easy to see how the reliable identification of patterns that consist of the interplay of multiple clues can quickly become overwhelming for a human analyst. To take all these aspects into account, *DISCOVER* provides an API that allows the user to define a set of rules that are used to automatically identify scenes based on previously defined conditions. Building upon the results of the visual inspection workflow, see Section 5.3, a user can now follow a blueprint script to download the smile detection annotations for both roles from the database, move a sliding window over the annotations and create a new annotation based on a predefined mirroring condition, see Figure 6.

## 6 User study

In this section we want to take advantage of all the above-mentioned capabilities of *DISCOVER* and its associated possibilities to present an application based on a user study that was conducted with prospective psychotherapists. In this study, *DISCOVER* summarized and interpreted key information from clinical interviews with patients to help psychotherapists plan their treatment in the form of a case conceptualization. Initial findings from this study will be presented.

### 6.1 Motivation

Personalizing treatment to the individual needs of the patient is an integral part of psychological therapies. While therapeutic decisions are guided primarily by intuition, there are now increasing efforts to integrate evidence-based and data-based recommendations into the therapeutic decision-making process (51, 52). Research in this area has traditionally focused on two main areas: Recommendations at the start of treatment and signals on adaptations during treatment if the desired outcome of therapy is not achieved (53).

This study explored a third, less examined area, namely the development of case conceptualizations (54). Case conceptualizations conclude the clinical assessment phase and aim to develop an individual model of the development and maintenance of the patient’s disorder. Based on a specific theoretical framework, they inform a treatment plan that initiates therapeutic change processes such as behavioral activation or restructuring maladaptive core beliefs (55). As expected, the quality of the case conceptualization is largely determined by the quality of the clinical assessment and the information derived from it (54). Furthermore, the development of such models seems to be strongly influenced by clinicians’ training and experience (56).

The Transtheoretical Treatment and Training Model (4TM; (57)) provides a theoretical foundation for the development of case conceptualizations by offering a structured framework for understanding and representing mechanisms of psychological change. It supports the training of prospective therapists by helping them identify clinically relevant features of patient experience and formulate hypotheses about underlying change processes to guide intervention planning. In the user study, we applied the 4TM model by integrating it into the *DISCOVER* framework, enabling the use of innovative data-informed methods to personalize case conceptualizations. By leveraging artificial intelligence within this framework, we analyzed both verbal and non-verbal aspects of initial clinical interviews, which typically serve to collect a broad range of information on the patient’s experience. These analyses formed the basis for tailored feedback provided to prospective therapists.

However, this study did not focus on evaluating the effectiveness of this feedback to personalize treatment, but rather on investigating how future users (prospective therapists) interact with the *DISCOVER* framework and evaluate the provided feedback. Research has shown that adapting training and design to the needs of users is a critical prerequisite for the successful implementation and sustainable integration of such tools into clinical practice (51, 58).

Initial clinical interviews are conducted to gather essential information, such as current symptomatology, biographical background, and treatment expectations. At the same time, therapists gain an emotional impression of the patient. For instance, if a patient talks about a pleasant mountain hike but does not display corresponding positive emotions such as a smile on their face or a lively tone of voice, this incongruence may indicate underlying psychopathology. However, therapists often have to carry out multiple tasks simultaneously, such as taking notes, tracking conversation content and deriving potential hypotheses, which may result in important information being lost (59, 60). Therefore, this study assisted prospective therapists by analyzing verbal, paraverbal and non-verbal aspects of clinical interviews using artificial intelligence within the *DISCOVER* framework.

Fully integrated into the 4TM, the study consists of two methodologically distinct parts: First, a multimodal analysis was used to identify emotionally salient scenes, which were then evaluated by users in terms of emotional importance and congruence. The second part investigated the integration of *LENS* into the 4TM. Users were presented with LLM-generated responses to characteristic questions about the patient’s experience and rated the appropriateness of these responses. Finally, users interacted with *LENS* independently and verbalize their thoughts as part of a think-aloud protocol.

The study aims to evaluate the potential of this data-informed approach to case conceptualization from the perspective of future users, i.e., prospective therapists. This approach supports prospective therapists in structuring and validating their case conceptualizations by ensuring that key information is considered and systematically assigned to the relevant facet of the patient’s experience. In doing so, it reduces cognitive load, highlights clinically relevant signals, and fosters reflective clinical reasoning.

The following section outlines the methodological setup of the study, including the selection of scenes, the LLM prompting process, and the integration of study components into a survey designed to guide users during tool interaction.

### 6.2 Scene selection

In the context of the scene selection process, a multimodal emotion analysis was conducted within *DISCOVER* on videos of simulated clinical interviews. The study was built on the work of Eberhardt et al. (61), which demonstrated associations between sentiment analysis and significant psychotherapeutic processes as well as therapeutic outcome. Consequently, written transcripts of clinical interviews were automatically generated and speaker diarization was applied within *DISCOVER*. These transcripts served as the basis for analyzing the patients sentiment. To achieve a robust estimation, the output of three sentiment analysis models were combined: The Multilingual Language Model Toolkit for Sentiment Analysis [XLM-T; (34)], the German Sentiment Classification Model [german-sentiment-bert; (35)], and LLM-based sentiment analysis through *LENS* using Mistral NeMo 12B (62). The extracted sentiment scores, ranging from negative (−1) over neutral (0) to positive (+1), were used as a prerequisite for the subsequent multimodal classification of intrapersonal affective (a)synchrony. Consequently, facial emotion recognition models [EmoNet; (42), LibreFace; (39), RBDM; (43)] and a vocal emotion detection model [Emow2v; (36)] were applied to the video and audio recordings of the clinical interviews. The models predicted, among others, eight categorical emotions (EmoNet, RBDM, LibreFace) and continuous valence (range 0 to 1; EmoNet, RBDM, Emow2v). Each transcript segment was divided into one-second intervals, and model outputs were aggregated within each interval to determine the dominant sentiment, categorical emotion, and valence. This allowed for the detection of subtle emotional shifts even within longer speech segments. Majority voting was then used to determine the dominant value in each class. *Affective asynchrony* was classified if the verbal sentiment and the non-verbal signals (categorical emotions or valence) did not match. Consequently, *affective synchrony* was detected if the verbal sentiment was consistent with the non-verbal signals. The sentiment value of ±0.5 served as a threshold for both classifications, i.e., if the sentiment was positive (>0.5) and accompanied by a negative emotion and/or a valence below 0.5, the segment was classified as affective asynchrony. If the emotion was positive and/or the valence was above 0.5, the segment was classified as affective synchrony. The automatic process generated time-stamped scene annotations for affective (a)synchrony in three categories: emotion, valence, and both. This resulted in scenes where either the categorical emotion or the valence rating, or both, were (a)synchronous. Annotations were stored in *DISCOVER* and selected for evaluation in the study if the corresponding speech segments were at least five seconds long and classified as *both* for at least 70% of the duration. From these, three scenes were randomly selected for affective synchrony and three for affective asynchrony.

### 6.3 Interaction with *LENS*

In the second part of the study, users were presented with LLM-generated responses to characteristic questions about the patient’s experience. The interaction with the LLM was carried out by the authors of this study as part of the study preparation. Specifically, for five of the eight facets of the 4TM (Emotion, Cognition, Behavior, Motivation and Interpersonal), the psychologists among the authors developed five questions each. These facets were selected to ensure feasibility within the study’s limited time frame while still covering a broad range of clinically relevant domains. The questions were then incorporated into prompts containing the automatically generated transcripts of the clinical interview and directed to the LLM-assistant using the model Mistral NeMo 12B separately for each patient. See Supplementary Figure S1 for an overview of the prompt architecture and Supplementary Table S1 for the specific questions that operationalize the five facets of the 4TM. The responses of *LENS* were then used in another prompt to rate the burden of each facet for the individual patient on a scale of 1 (*low burden*) to 3 (*high burden*) by the same LLM. Facets that were rated as highly burdened by *LENS* could be selected by the users in the study for further exploration, i.e., the answers to the five questions of that facet were presented to the therapists, who were asked to rate the fit of the answers and to give their reasons if the fit was rated as poor. In the final part of the study, users had the opportunity to ask *LENS* their own questions. In a think-aloud approach, they were encouraged to share their rationale for the questions and to match their questions to one of the facets of the 4TM. This part of the study was audio recorded to capture users’ verbal reasoning.

### 6.4 Procedure

**Figure 7:**
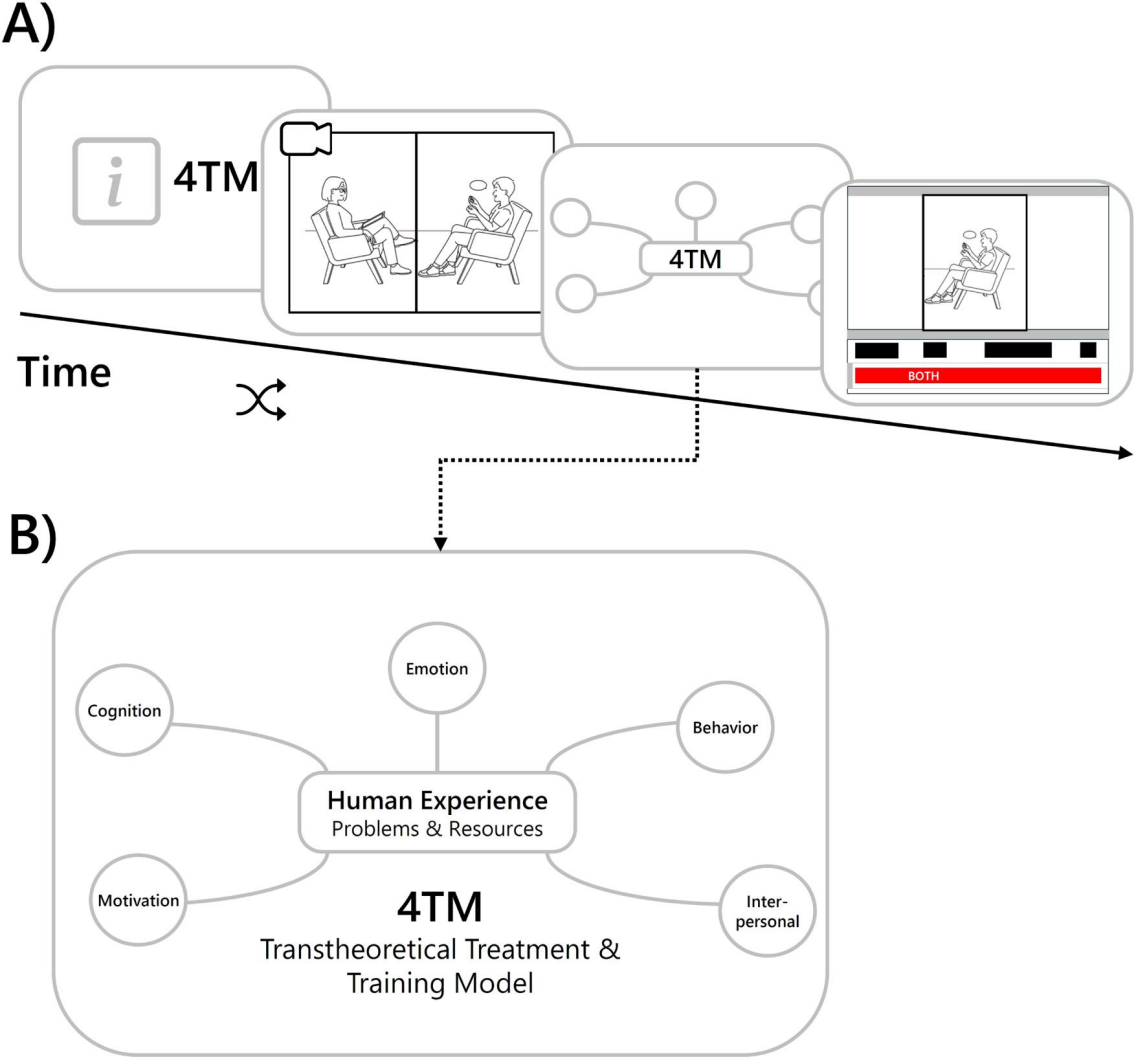
Integration of the Transtheoretical Treatment and Training Model into the DISCOVER framework. (**A**) Visualization of the tool interaction. Drawing from: “Stock photo of man, talking, psychologist”, licensed under royalty-free vector. Info square icon from: “Info square”, licensed under MIT license. (**B**) Graphical representation of the 4TM model. Adapted with permission from “Core Elements of a Transtheoretical Treatment and Training Model (4TM)” by Lutz et al., licensed under CC BY 4.0.

**Figure 8:**
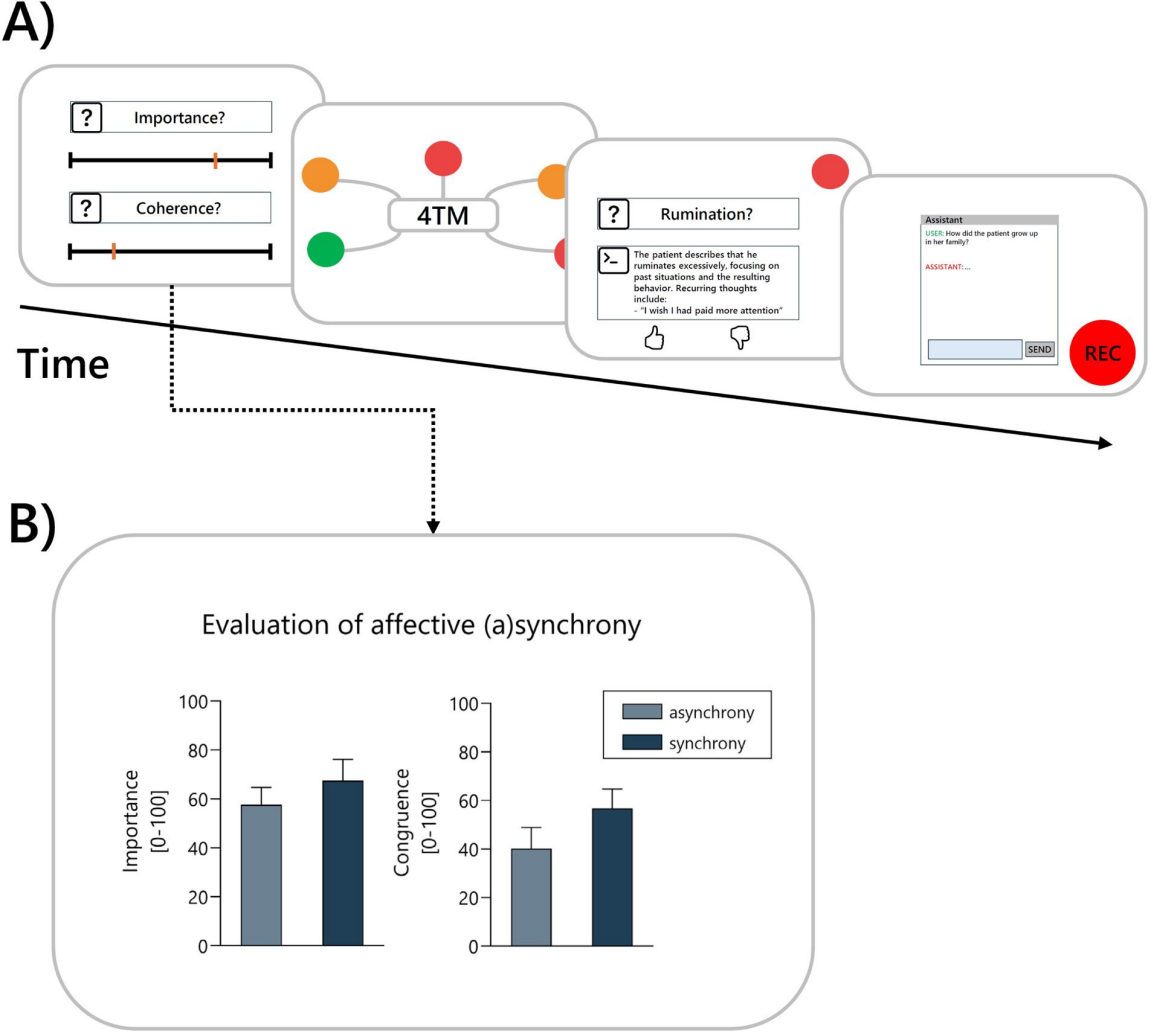
User evaluation of outputs generated within the *DISCOVER*  framework. **(A)** Visualization of the evaluation procedure. **(B)** Initial findings from the user evaluation of the scenes selected from multimodal emotion analysis.

At the beginning of each study session, prospective therapists were introduced to the 4TM and the five facets of human experience that were central to the study. They were then randomly assigned to one of four simulated clinical interviews, which they were asked to watch carefully while taking notes. The videos were available for one-time viewing only and could not be paused, in order to provide therapists with conditions comparable to those of real clinical interviews. The therapists were then presented with a graphic representation of the corresponding facets of the 4TM (see Figure 7B). They were asked to imagine that they were treating the portrayed patient and to rate the degree of burden for the depicted facets by coloring them according to the coloring scheme: green = *low burden*, orange = *medium burden*, and red = *high burden*. The focus was then placed on the emotional facet and the therapists were again confronted with scenes from the clinical interview in which intrapersonal affective asynchrony had been identified by the scene selection algorithm described above (see Figure 7A) for an overview of the tool interaction procedure. After each scene, the therapists were asked to rate its importance for understanding the patient’s emotional experience and the perceived congruence between verbal and non-verbal signals on visual analogue scales ranging from *not at all* to *very much*. A total of six scenes (three synchronous and three asynchronous) were presented to the therapist in randomized order. At the end of the task, therapists were asked to provide an overall assessment of the selected scenes. On a five-point Likert scale, they were asked to rate the helpfulness of the selection from *very helpful* to *not helpful*. In addition, they were asked whether any important scenes were missing and, if so, which ones. In the next part of the study, therapists were again presented with the graphical representation of the 4TM model, but this time colored according to the *LENS*’s ratings of patient burden. They were then asked to successively select two of the red facets (*high burden*) and rate the LLM-assistant’s answers to the corresponding questions. The rating was a binary choice (thumbs up or down) and negative ratings (thumb down) needed to be explained. In the final part of the study, the therapists interacted with *LENS* while being recorded via audio. Each question should be assigned to a facet of the 4TM and therapists were asked to articulate their reasoning. A detailed visualisation of the evaluation procedure is provided in Figure 8A.

The following section presents initial findings from the first part of the user study, which was conducted on a small sample of prospective psychotherapists.

### 6.5 Sample description

Twelve prospective psychotherapists participated in the user study. They were recruited either by notices posted at the training institute or via email. On average, the users were 29.75 years old (*SD* = 3.17), with 50% identifying as female. At the time of the study, they were in their sixth semester of training (*M* = 5.5, *SD* = 3.1) and were treating a mean of 7.58 (*SD* = 4.65) outclinic patients.

### 6.6 Initial findings

The initial findings reported here are based on the first stage of the study, in which users were presented with the results of the multimodal emotion analysis and the subsequent scene selection process. Users evaluated emotionally salient scenes in terms of their emotional importance and the congruence between verbal and non-verbal affective expressions. As expected, scenes that had been classified as asynchronous by *DISCOVER* (i.e., showing a mismatch between verbal and non-verbal affect) were perceived as being less congruent (*M* = 40.22, *SD* = 25.23) by the users than scenes that had been classified as synchronous (*M* = 56.72, *SD* = 23.39). These results suggest that the models implemented in *DISCOVER* are sensitive to variations in affective synchrony. Furthermore, asynchronous scenes were rated as less important to the therapist’s understanding of the patients emotional experience (*M* = 57.61, *SD* = 20.76) than synchronous scenes (*M* = 67.53, *SD* = 25.41). This hints at the fact that perceived affective (a)synchrony influences the therapist’s assessment of the emotional relevance of therapeutic interactions. A visualization of these findings is provided in Figure 8B. On an overall level, the presented scenes received an average rating of 2.42 (*SD* = 0.86) on a five-point Likert scale (1 = *very helpful* to 5 = *not helpful*), indicating that they were perceived as rather helpful. At the same time, 75% of users stated that important scenes were missing, which is understandable given that each user was only presented with six emotionally significant scenes selected from the clinical interview.

## 7 Discussion

With *DISCOVER* we presented a novel, modular, and flexible framework designed to lower the entry barrier for computational behavior analysis. By integrating audio, video, and text processing capabilities into a unified framework, *DISCOVER* facilitates multimodal analyses for researchers across varying levels of technical expertise. A central component of this framework is *LENS*, a natural language interface that uses large language models to allow the interactive creation of annotations and the exploration of semantic content based on the transcript of a user interaction. The user study included in this work demonstrates how *DISCOVER* can effectively support clinical training of psychotherapists by transforming complex behavioral data into actionable insights. Specifically, we demonstrated how the system can be used to identify nuanced intrapersonal characteristics, such as affective (a)synchrony, to identify salient scenes during psychotherapy sessions. Furthermore, we showed how *LENS* can be employed to assist prospective psychotherapists with case conceptualizations. This capability has the potential to enhance clinical training by supporting the systematic organization and prioritization of patient information, thereby helping prospective therapists to focus on the most relevant aspects for case conceptualization.

Current tools for analyzing human interaction (see Section 2) are equipped with functionalities to gather new information, but often present limitations in terms of accessibility, adaptability, and data integration. Historically, these systems have relied heavily on manual annotation, a process that, while capable of high accuracy when meticulously applied, is inherently time consuming, resource intensive, and ill-suited for large datasets or complex interaction scenarios. The sheer volume of data generated in contemporary research makes manual annotation increasingly impractical. Furthermore, automated annotation tools often lack the versatility required for broad application, remaining constrained by specific use cases or data modalities. In contrast, *DISCOVER* provides a unified platform capable of simultaneously processing and analyzing multimodal data streams.

Furthermore, existing tools often lack the flexibility to incorporate new analytical techniques or integrate novel machine learning models. Although some tools offer scripting capabilities, these are often limited and require a good understanding of the underlying software architecture. *DISCOVER*, on the other hand, is built on a modular architecture that allows for a straightforward integration of custom modules and algorithms. This enables researchers to leverage the latest advances in machine learning and artificial intelligence to address complex research questions. The natural language interface *LENS*, also distinguishes *DISCOVER* from many existing tools. *LENS* allows researchers to query the data using natural language, allowing a more exploratory and iterative approach to data analysis. This can be particularly valuable in the early stages of research, when the researcher may not have a clear understanding of the patterns and relationships within the data. This exploratory capability empowers researchers to formulate new hypotheses and refine their research questions. Finally, *DISCOVER* utilizes a server-based architecture, providing researchers with unified access to computational resources. This centralized approach streamlines data processing and analysis, reducing the need for individual infrastructure and promoting collaborative research efforts.

Despite its advantages, *DISCOVER* is not without limitations. The accuracy of automatic annotations and predictions is inherently dependent on the quality and diversity of the underlying models and training data. Biases in these models can lead to misinterpretations, especially when analyzing culturally specific behaviors or working with underrepresented populations. For instance, a model predominantly trained on data from Western cultures may misread non-verbal cues common in Eastern cultures. Particularly, because *DISCOVER* is aimed at non-experts in the technical domain, it is important to make these limits transparent to the user. Furthermore, while the system is designed for user-friendliness, initial setup and the integration of custom modules may still require technical support. Reliance on a cloud-based or centralized processing infrastructure also raises concerns regarding data privacy and compliance with institutional or legal requirements.

## 8 Conclusion

We presented *DISCOVER*, a novel system designed to address persistent challenges in the analysis of behavioral data, particularly within the domain of social interaction. By integrating a flexible and extensible annotation framework with automated data processing capabilities, *DISCOVER* offers a substantial advancement over traditional methods reliant on manual coding, which are often time-consuming, resource-intensive, and prone to subjective biases.

The integration of various state-of-the-art modules for audio and video analysis, as well as the incorporation of LLM-based semantic exploration and sentiment analysis, equips researchers with a robust suite of tools to conduct in-depth analyses without requiring extensive technical expertise. Additionally, by emphasizing interactive data exploration through features like visual inspection, aided annotation, and scene search, discover makes it easier to extract complex behavioral patterns, which improves the breadth and accuracy of social science research. We presented initial findings of a user study with prospective psychotherapists, designed to evaluate the potential of the *DISCOVER* framework in supporting case conceptualization. The study focused on its usability in clinical practice, particularly with regard to structuring psychological assessments and enabling data-informed treatment planning.

However, the development and deployment of automated behavioral analysis systems are not without challenges. A critical consideration is the potential for algorithmic bias. The accuracy and generalizability of automated schemes depend heavily on the quality and representativeness of the training data. If the training data are skewed towards certain demographics or behavioral expressions, the system may exhibit reduced performance or even systematic errors when applied to different populations. Therefore, rigorous validation and ongoing monitoring are essential to ensure fairness and avoid perpetuating existing societal biases. Another challenge lies in the inherent complexity of human behavior. While automated schemes can effectively identify predefined behaviors, capturing the full richness and context of social interactions requires sophisticated algorithms capable of handling ambiguity, variation, and individual differences.

Looking ahead, there are a number of particularly promising directions for future study. Expanding the library of annotation schemes and automated prediction models to encompass a wider range of behavioral cues, including gaze patterns, vocal characteristics, and body posture, would significantly enhance the system’s versatility. By empowering researchers with a versatile tool for unlocking deeper insights into the complexities of human behavior, *DISCOVER* has the potential to substantially advance our understanding of social interaction and its impact on human well-being.

## Data Availability

The data analyzed in this study is subject to the following licenses/restrictions: GDPR. Requests to access these datasets should be directed to tobias.hallmen@uni-a.de.

## Footnotes

^1^ https://github.com/hcmlab/discover

^2^ https://github.com/hcmlab/nova

^3^ https://www.mongodb.com

^4^ https://nextcloud.com

^5^ https://chatgpt.com

^6^ https://ollama.com

^7^ https://github.com/hcmlab/discover-modules

^8^ https://huggingface.co/
