# Supplementary material for: DISCOVER: a Data-driven Interactive System for Comprehensive Observation, Visualization, and ExploRation of human behavior
Source: Front Digit Health. 2025 Sep 19;7:1638539. doi: 10.3389/fdgth.2025.1638539 (PMC12492635; doi:10.3389/fdgth.2025.1638539)
Supplement: Supplementary file 1 [file Datasheet1.pdf]

Supplementary Material

1 TRANSCRIPT

The entire conversation dialogue utilized in the interactive data exploration workflow is presented here. The transcript has been anonymized and translated into English. No corrections have been made to the grammatical or lexical errors introduced by the transcription module. The segmentation into speaking segments remains unchanged from the output provided by the transcription module. The comments in square brackets were added by the author to facilitate a better understanding.

TEACHER: Ah, good morning, Mrs. Doe.

PARENT: Good morning.

PARENT: It's great that it works.

TEACHER: So, first of all, the technology works. [The conversation is held via Zoom]

TEACHER: Can you hear me well?

PARENT: Yes, I see you and I hear you.

TEACHER: Do you see me well?

TEACHER: Does everything work?

TEACHER: Okay, super.

TEACHER: Then I would like to talk to you right away.

TEACHER: We now have roughly five to eight minutes to talk about your request.

TEACHER: And I would ask you first of all, why did you want to talk to me today?

PARENT: We are very, very worried about the e-mail [Emil] right now.

PARENT: I have to say that since he has been in your class in his fourth year, we no longer like his grades.

TEACHER: Mhm.

TEACHER: Mhm.

PARENT: I thought he's done a great job so far.

PARENT: He was always kind of on a solid two and now he's kind of slipping this school year.

PARENT: And it's always such a close call.

PARENT: It always sloshes so close to the two.

PARENT: And that really stresses us out because he's supposed to go to grammar school.

PARENT: Or what means suppoeds to... he wants that too.

PARENT: So he wants to do his A-levels himself and then go to university.

PARENT: That's really important to him.

PARENT: And it doesn't look like it's going to work out with the grammar school.

PARENT: And I don't know what we can do or what you can do, because it's only since this school year, since he's been with you, that things have stopped working.

TEACHER: Okay.

PARENT: Why?

TEACHER: Okay, so I can hear that you're very worried about Emil, especially now with regard to the transfer, because we're already in February and the transfer is due in May.

PARENT: Absolutely.

TEACHER: I can definitely understand that and do I understand correctly that Emil was actually always quite good in third grade and his grades have only now slipped down to a three and a four in fourth grade, right?

TEACHER: Yes.

TEACHER: Okay.

TEACHER: Yes, as I said, I understand that you're worried.

TEACHER: And I would now like to ask you what you would like for Emil now, especially with regard to the transition?

TEACHER: What do you want for Emil?

PARENT: I hope he makes the transition to grammar school.

PARENT: So that the grades are good, that I have my happy Emil again, who comes home and is somehow doing well and where the school day went well.

TEACHER: Okay.

PARENT: He's also kind of stressed at the moment and when we talk about school, it's so tense.

PARENT: He also doesn't enjoy it as much because he doesn't have the success he wants for himself.

PARENT: And I hope that he is doing well and that the grades are right.

TEACHER: Okay, so they just want him to be a bit less stressed when he transfers to grammar school.

PARENT: Yes.

TEACHER: That's the wish.

TEACHER: Okay, so I would like to start by describing my situation.

TEACHER: Of course, I can also see that Emil's performance is rather mediocre at the moment, and that he's not doing so well.

TEACHER: Yes, it's a bit difficult with the GÜMI at the moment, with the editing.

TEACHER: But I can put your mind at rest for now.

TEACHER: If Emil really doesn't make it to grammar school at the end of May, there is always the option of transferring to grammar school at the end.

TEACHER: So even if he really doesn't make it with this average of 2.33, you don't have to worry that the Abitur and everything will be ruined straight away, because it's not like that.

TEACHER: So you can still...

TEACHER: once you're at secondary school, if you have a good average of 2.0, for example, then you can always switch back to grammar school.

TEACHER: So this step with the Abitur is not blocked just because Emil doesn't go straight to grammar school after the fourth grade.

PARENT: Oh, actually, okay.

PARENT: Oh, we weren't really aware of that, okay.

PARENT: Because my husband and I both went down that route, somehow through grammar school and on to A-levels.

PARENT: Oh, okay.

PARENT: Theoretically, that would still be possible.

TEACHER: Okay, and then, yes, as I said, I can understand that it's a bit frightening at first if it doesn't work out straight away, but we still have time.

PARENT: Mhm.

TEACHER: It's only February now and we still have a few months until the transition and maybe things will improve then.

PARENT: Yes, three.

TEACHER: I'd like to know what you do with Emil at home to maybe support him a little, right?

PARENT: So we practise a lot.

TEACHER: Mhm.

TEACHER: Mhm.

PARENT: So when he comes home, we have lunch and then he actually sits down at the driving table and does his things.

TEACHER: Mhm.

PARENT: That means his homework and I also check in every now and then if he needs help somewhere or wants to make sure that everything is working properly.

PARENT: And then ask him a lot of questions.

PARENT: So when he's finished with his homework, we always look at the HSU booklet again and go through the things in this notebook if they've made any new entries, so that we can repeat them.

PARENT: And yes, we practise one thing very, very regularly and all those big numbers somehow, which they have in fourth grade right now.

TEACHER: mhm mhm

PARENT: So we practise super, super much.

PARENT: My husband, even when he comes home from work, goes through things with him again.

PARENT: So we really do a lot.

PARENT: And that's why these notes are totally baffling to me right now.

PARENT: So he can do that at home.

TEACHER: Okay, I can understand.

TEACHER: So now I understand that you really practice a lot with Emil at home and that you get right behind him every day and practice a lot, especially now in math with Emil.

PARENT: Yes

TEACHER: So for me personally it would be important. . .

TEACHER: Of course you can support your child at home, but don't overwhelm them at home.

TEACHER: So leave the child alone sometimes, maybe half an hour, an hour a day, that's all right, but don't sit him down at lunchtime and sit at the table with the child for three hours, because the child's well-being is of course the most important thing.

TEACHER: And the child should enjoy and succeed in learning, that's super important.

PARENT: Yes, but if we don't step on the gas now, he won't be successful.

TEACHER: So I would now offer to really pay close attention in class to how things are going with Emil, also in terms of concentration, to see if there is perhaps a problem with concentration or motivation.

TEACHER: I would now pay explicit attention to this again in class and take a close look at the Emil over the next few weeks.

TEACHER: And I would ask you to really just, yes, support Emil at home, but make sure that he really still has fun doing it.

TEACHER: And if you notice that he has lost concentration and is unmotivated, then leave it alone for the day.

TEACHER: Yes. You can really do that.

TEACHER: I still notice that you are a bit insecure.

TEACHER: But that's also my job at school, to encourage Emil and to make sure that Emil is always focused and motivated and that he enjoys what he's doing.

PARENT: You really think we should do a little less.

PARENT: But won't that make us slip even more?

TEACHER: So if that's okay with you, I would try it out for a few weeks.

TEACHER: We can start in two or three weeks, so that you can relax a bit at home and really pay attention to how long his concentration lasts and if he really falls off completely and is unmotivated, that you simply say, okay, now it's good and I'll pay attention in class to how things are going and then we can talk again in two or three weeks and we could talk about his developments and progress.

PARENT: Yes.

PARENT: Okay, okay, good.

TEACHER: Is that okay with you?

PARENT: Yes, it's such a short period of time, you can try it out for two weeks somehow.

PARENT: I still have a bit of a stomach ache, but if you say that it might take a bit of pressure off, that it might be a solution or help us all a bit, then okay, let's give it a try.

TEACHER: Okay.

TEACHER: Mhm, ja.

TEACHER: Mhm.

TEACHER: Exactly, so that would be a solution to start with and then we'll just get in touch in two weeks or we can also do three weeks.

PARENT: Ja.

PARENT: Ja.

PARENT: Ja.

TEACHER: Let's get in touch again and then we'll talk about it again in a longer meeting.

TEACHER: Then we'll take a little longer and maybe have a chat with Emil the three of us together.

PARENT: Ah, okay, me, me, okay.

TEACHER: Exactly, that's what I would suggest now, if that's okay with you.

PARENT: Yes, if you say that this might be a solution or a bit of relaxation, maybe a good idea now, okay, you're the expert, then let's give it a try.

TEACHER: Yes.

TEACHER: Exactly, that would be great.

PARENT: Okay

TEACHER: Let's try it out at the beginning and then we'll talk again in two weeks.

TEACHER: I will then get back to you.

PARENT: Okay, good, good, thank you very much.

PARENT: Yes, well, let's give it a try.

TEACHER: Yes, with pleasure, Mrs. Doe.

TEACHER: Goodbye.

PARENT: Thank you.

PARENT: Goodbye.

TEACHER: Goodbye.

2 LENS INTERACTION

The full interaction of a user with LENS for the *Interactive Semantic Content Exploration* Workflow:

1.

USER: Summarize the transcript

LENS: The transcript is a conversation between a teacher and a parent (Mrs. Doe) about their child, Emil, who is struggling in school. The parent expresses concern that Emil's grades are not improving despite their efforts to help him at home.

The teacher listens attentively and acknowledges the parent's concerns. She suggests that maybe they are overdoing it with homework and that it would be beneficial for Emil to have some downtime and freedom to enjoy learning without feeling pressured or stressed.

The teacher offers to work closely with Mrs. Doe to monitor Emil's progress and adjust their approach as needed. She proposes a trial period of two weeks where they will focus on supporting Emil's learning while also giving him space to relax and recharge.

Mrs. Doe agrees to try this approach, acknowledging that it might be helpful for Emil to have some relief from the pressure to perform well academically. The teacher reassures her that she will follow up with Mrs. Doe in two weeks to discuss Emil's progress and make any necessary adjustments.

Overall, the conversation is a collaborative effort between the teacher and parent to find a solution that supports Emil's learning and well-being.

2.

USER: What are important criteria to assess the quality of a dialouge in the setting of a parent-teacher conference?

LENS: When assessing the quality of a dialogue in the setting of a parent-teacher conference, consider the following important criteria:

1. **Mutual understanding**: Do both parties (parent and teacher) demonstrate an understanding of each other's perspectives, concerns, and goals?

2. **Open-mindedness**: Are both parties open to listening to each other's views, even if they differ from their own?

3. **Active listening**: Is the dialogue characterized by active listening, where both parties focus on the speaker, ask clarifying questions, and respond thoughtfully?

4. **Respectful tone**: Is the dialogue conducted in a respectful tone, free from blame, criticism, or defensiveness?

5. **Collaborative approach**: Does the dialogue foster a collaborative approach to problem-solving, where both parties work together to find solutions?

6. **Clear communication**: Are the messages conveyed by each party clear, concise, and easy to understand?

7. **Empathy and understanding**: Do both parties demonstrate empathy and understanding for each other's emotions, concerns, and experiences?

8. **Proactive problem-solving**: Does the dialogue lead to proactive problem-solving, where both parties work together to address issues and find solutions?

9. **Follow-up and follow-through**: Are there clear plans for follow-up and follow-through on agreed-upon actions or next steps?

10. **Positive outcome**: Does the dialogue result in a positive outcome, such as increased understanding, improved communication, or a plan for addressing concerns?

By considering these criteria, you can assess the quality of the dialogue and determine whether it has led to a productive and effective parent-teacher conference.

3.

USER: Analyze the transcript with respect to those criteria.

LENS: Based on the transcript, I will analyze it with respect to the following criteria:

1. **Turn-taking**: The conversation is characterized by a clear turn-taking pattern, where the teacher and parent take turns speaking. The teacher initiates most of the turns, but the parent also contributes significantly.

2. **Topic development**: The conversation starts with the parent's concern about their child's (Emil) poor grades and lack of motivation. The topic is developed throughout the conversation, with the teacher offering suggestions and advice to support Emil's learning.

3. **Questioning**: The teacher asks several questions to clarify the situation and understand the parent's concerns. The parent also asks a few questions in response.

4. **Active listening**: Both the teacher and parent demonstrate active listening by acknowledging each other's statements and showing understanding.

5. **Empathy**: The teacher shows empathy towards the parent's concerns and frustrations, which helps to build trust and rapport.

6. **Problem-solving**: The conversation focuses on finding solutions to support Emil's learning. The teacher offers suggestions for the parent to try at home, such as reducing homework time and encouraging breaks.

7. **Collaboration**: The teacher and parent collaborate to find a solution that works for both parties. They agree to work together and re-evaluate their approach in two weeks.

Some specific observations:

- The conversation starts with a clear statement of the problem (Emil's poor grades) and then explores possible causes and solutions.
- The teacher uses open-ended questions to encourage the parent to share more information about Emil's learning habits and challenges.
- The parent is receptive to the teacher's suggestions and ideas, which suggests that they are willing to work together to find a solution.
- The conversation is characterized by a calm and respectful tone, with both parties showing understanding and empathy towards each other.

4.

USER: What are some non-verbal signs to look out for to assess the quality of the dialogue?

LENS: When assessing the quality of a dialogue, here are some non-verbal signs to look out for:

1. **Body Language**: Pay attention to the parent's and teacher's body language:
    - Are they leaning forward or backward, indicating engagement or disinterest?
    - Are their arms crossed, suggesting defensiveness or closed-offness?
    - Are they maintaining eye contact, showing interest and attentiveness?
2. **Facial Expressions**: Note the facial expressions of both parties:
    - Are they smiling, indicating a positive tone and rapport?
    - Are their eyebrows furrowed, suggesting concern or skepticism?
    - Is there a neutral or flat expression, indicating a lack of emotional investment?
3. **Proximity and Distance**: Observe how close or far apart the participants are:
    - Are they sitting at a comfortable distance, indicating mutual respect?
    - Are they leaning in or standing closer, suggesting intimacy or emphasis?
4. **Posture and Alignment**: Pay attention to their posture and alignment:
    - Are they sitting up straight, indicating confidence and openness?
    - Are they slouching or leaning away, suggesting discomfort or disinterest?
5. **Touch and Physical Contact**: Note any physical contact or touch between the participants:
    - Is there gentle touching or patting on the back, indicating empathy and support?
    - Are they avoiding physical contact, suggesting a lack of comfort or trust?
6. **Eye Contact and Gaze**: Observe how long each participant maintains eye contact with the other:
    - Are they maintaining steady eye contact, indicating engagement and interest?
    - Are their gazes darting away, suggesting discomfort or distraction?
7. **Vocal Inflections and Tone**: Pay attention to the tone and inflection of their participants

3 4TM MODEL

Table S1. Overview of the specific questions that operationalize the five facets of the 4TM.

4TM Facet	Example Questions (translated from German)
Emotion	1. How would you describe the patient's general mood during the session?<br>2. What are the central emotions expressed?<br>3. What does the transcript reveal about emotion regulation?<br>4. Can the patient allow and express emotions?<br>5. How would you describe the patient's affect in psychopathological terms (according to the AMDP system)?
Cognition	1. What distressing thoughts or core beliefs does the patient express during the session? Please include direct quotes if possible.<br>2. Can you identify any typical cognitive distortions or thinking errors in the patient's statements, particularly according to Aaron Beck's theory?<br>3. Are there any indications that the patient is engaging in excessive rumination?<br>4. What topics or problems is the patient preoccupied with?<br>5. How would you describe the patient's formal thinking during the session in psychopathological terms (according to the AMDP system)?
Behavior	1. What problematic behaviors does the patient report during the session?<br>2. Are there any indications of pronounced avoidance or social withdrawal?<br>3. Does the patient report signs of lack of motivation or drive during the session?<br>4. Is the patient engaged in positive activities, interests, or hobbies, or are there signs of a reduction in these areas?<br>5. What has the patient done so far to cope with their problems?
Motivation	1. Does the patient express motivation or willingness to change?<br>2. What motives and needs does the patient articulate, and how do these become apparent during the session?<br>3. What goals does the patient formulate for therapy or for life more generally? What does the patient hope to gain from psychotherapy?<br>4. Is the patient willing to engage emotionally and personally in therapy and to share their inner experiences?<br>5. Are there indications of ambivalence regarding therapy or specific problem areas?
Interpersonal	1. Are there any noticeable interpersonal behaviors displayed by the patient during the session?<br>2. Does the patient describe any striking interpersonal behavior outside of the therapy session?<br>3. How would you describe the therapeutic relationship between the patient and the therapist?<br>4. Are there signs of ruptures or tensions in the therapeutic alliance (so-called "alliance ruptures")?<br>5. How would you classify the patient's behavior using the interpersonal circumplex (Kiesler Circle)? Does the patient tend to show friendly or hostile, dominant or submissive tendencies?

```
#System prompt
"You are a therapeutic assistant helping me analyze transcripts from
   psychotherapy sessions."

#User prompt
f"Analyse the following transcript:
     \"\"\"
     {transcript}
     \"\"\"
{question}"
```

Figure S1. Sample code demonstrating how to create an annotation for detecting smile mirroring.
